# Respiration characteristics and its responses to hydrothermal seasonal changes in reconstructed soils

**DOI:** 10.1038/s41598-020-80623-4

**Published:** 2021-01-08

**Authors:** Na Lei, Juan Li, Tianqing Chen

**Affiliations:** 1 Shaanxi Provincial Land and Engineering Construction Group Co., Ltd., Xi’an, Shaanxi China; 2 Institute of Land Engineering and Technology, Shaanxi Provincial Land Engineering Construction Group Co., Ltd., Xi’an, Shaanxi China; 3 Institute of Soil and Water Conservation, State Key Laboratory of Soil Erosion and Dryland Farming on the Loess Plateau, Northwest A&F University, Yangling, Shaanxi China

**Keywords:** Agroecology, Climate-change ecology, Agroecology

## Abstract

Seasonal changes in respiration and the components of four reconstructed soils (gravel + meteorite + lou; gravel + shale + lou; gravel + sand + lou; and gravel + soft rock + lou) in barren gravel land were monitored using the soil carbon flux measurement system. The results showed that (1) the monthly average respiration rate and the rates of the components in the four reconstructed soils were the highest in summer and lowest in winter. In winter, the monthly average respiration rates of the four reconstructed soils were not different (p > 0.05). In summer, the monthly average respiration rate of the sand or meteorite reconstructed soil was different from that of the other three (p < 0.05). (2) The heterotrophic and autotrophic respiration rates were different between the four reconstructed soils (p < 0.05). The contribution of heterotrophic respiration to total respiration in the four reconstructed soils was greater than that of autotrophic respiration throughout the year. In winter, autotrophic respiration accounts for the smallest proportion of total respiration. As the temperature rises, the proportion of autotrophic respiration to total respiration gradually increases and peaks in summer. In summer, the proportion of heterotrophic respiration in the total respiration is the smallest. With the decrease in temperature, the proportion of heterotrophic respiration in total respiration gradually increases and peaks in winter. (3) The maximum and minimum values of the monthly average respiration rate of the four reconstructed soils coincided with the months of maximum and minimum soil temperature. The soil volumetric water content changed with the amount of precipitation. The correlation between soil respiration and temperature was greater than that between soil respiration and volumetric water content. (4) The correlation in seasonal variation between respiration of the four remodelled soils and hydrothermal factors in the study area can be characterised by an exponential function and power-exponential function.

## Introduction

Soil respiration and its minor changes significantly impact the atmospheric CO_2_ concentration, which in turn affects the global carbon balance^1^. The soil respiration rate exhibits strong temporal and spatial dynamic variability. The minimum rate of soil respiration is 60% of the maximum rate on a seasonal scale, and the day-night difference in the soil respiration rate can reach 5 g CO_2_ m^−2^ h^−1^ on a daily scale^2,3^. Therefore, in regional scale research, only the study of different seasonal changes in soil respiration and its influencing factors can lay a reliable foundation for the accurate estimation of soil CO_2_ emissions. Researchers believe that studying the seasonal dynamics of soil respiration is crucial for understanding the global carbon cycle process, and it provides data for monitoring interannual soil respiration changes. Therefore, the seasonal variation law of soil respiration is of wide concern.

The components of soil respiration include autotrophic respiration and heterotrophic respiration. The seasonal changes of soil respiration and its components are affected by temperature, moisture, other hydrothermal factors, and biological factors^4,5^. Most researchers believe that soil temperature is the main factor of seasonal changes^6,7^. There is a significant exponential relationship between soil respiration and soil temperature, and the response of farmland soil respiration to soil temperature is greater than that of grassland^8^. Some researchers believe that temperature and moisture together affect soil respiration^4^ when soil moisture conditions are adequate (water content was 25–40% by volume). A significant correlation has been shown between soil temperature and soil respiration; soil respiration was strongly inhibited when soil moisture was insufficient or excessive^9^. The components of soil respiration have seasonal variation characteristics similar to those of soil respiration^10,11^. The contribution of autotrophic respiration to total soil respiration is usually higher during the growing season and lower during the one-year dormancy period^12^. The contribution of autotrophic respiration to total respiration is less than that of heterotrophic respiration^11^; heterotrophic respiration has strong temporal and spatial variability. Organic carbon enters the atmosphere mainly through heterotrophic respiration and participates in the ecosystem carbon cycle process, affecting world climate change^13^. In addition, scholars have also studied the seasonal changes of soil respiration in different types of ecosystems, different land use types, different regions, and different time scales.

Barren gravel lands are widespread in Huayin, Baoji, and other places in Shaanxi Province. The existing projects for soil reconstruction were carried out by adding different soil materials and improved materials. The reconstructed soils will become the main approach in land remediation in the future and an important means to supplement cultivated land resources^14^. Concomitantly, the reconstruction of soils changes the changes the underlying surface conditions in the area^15^, resulting in altered soil respiration of the newly formed soils, which ultimately affects the regional climate. However, the studies on respiration of reconstructed soils are still rare. It is impossible to quantify the impact of reconstructed soils on climate change. With increasing economic and social development, the amount of reconstructed soils will increase exponentially^16^, and the impact on the climate will also increase. Research on the intensity of soil respiration of reconstructed soils will be useful for predicting and reducing atmospheric CO_2_ concentrations as well as the process of global warming; actions that are imperative for local governments and even countries. Therefore, it is urgent to study the seasonal changes in soil respiration and its components in reconstructed soils to quantify their impact on climate change; this is key to accurately predicting changes in soil respiration in the future. To address this, in the present study, four types of reconstructed soil supplemented with meteorite, shale, sand, and soft rock were selected as research objects. The characteristics of seasonal variation in respiration, hydrothermal factors of the four reconstructed soils, and the relationship between soil respiration and soil respiration composition were explored. The study provides a scientific reference and data that will support accurate assessment of regional CO_2_ emission and development of measures for CO_2_ reduction, which ultimately helps ensure ecological stability and safety.

## Materials and methods

### Experimental area

The experimental test plot is located in Meixian, which belongs to Baoji City, Shaanxi Province. It is located west of the Guanzhong Plain at the foot of Taibai Mountain, the main peak of the Qinling Mountains. The total area of 863 km^2^ has five landforms: Qinling Mountains, Loess Plateau, Huangtu Liangmao, Diluvial Plain, and Alluvial Plain. The area has a warm temperate continental semi-humid climate, with an average annual temperature of 12.9 ℃, and an average precipitation of 609.5 mm. The main soil type in Meixian is Lou soil. There are a large number of barren gravel lands in the experimental area, which have high gravel content, barren land, water and fertiliser leakage, and low vegetation coverage, which are not conducive to crop growth. It is necessary to rebuild cultivated land through soil reconstruction to increase the area of cultivated land and improve the regional ecological environment.

### Reconstruction materials

Up to 60 kinds of mineral materials have been studied to improve soil quality, and a series of improved products have been produced, such as mineral fertilisers, growth agents, and nutrient carriers. Mineral materials have natural and unique crystal structure and good physical and chemical properties of surface adsorption, ion exchange capacity, swelling, and acidity and alkalinity. They can improve soil structure, increase soil water retention capacity, increase soil fertility, adjust soil pH, and restore heavy metal pollution. Properly selecting mineral materials for soil reconstruction can produce better economic, ecological, environmental, and social benefits. The meteorite, shale, sand, and soft rock selected in this study are all mineral materials. Among them, soft rock and shale have high clay content as soil-forming materials. Meteorite, as an improvement material, can increase soil water retention. As a soil improvement material, sand can increase soil permeability. These four materials are distributed in Shaanxi Province and are easy to obtain.

### Overview of test plots

The test plot was located in Shangwang Village, Tangyu Town, Meixian County, Baoji City, Shaanxi Province (107°53′50″E, 34°8′33″N), in a demonstration area for the barren gravel land remediation project. The total area was 8.00 hm^2^, and the newly added cultivated land occupied 6.80 hm^2^. Four types of material, soft rock, sand, shale, and meteorite, were selected, crushed and sieved through a 10 mm sieve, disinfected, sterilised, and mixed with a prepared soil source to form a mixed layer (30 cm) of soil with meteorite, shale, sand, and soft rock. Lou soil, the local common soil type, was used in the experiment. Finally, four reconstituted soils were prepared, which were gravel + meteorite + lou, gravel + shale + lou, gravel + sand + lou, and gravel + soft rock + lou soil types (here after referred to as meteorite, shale, sand, and soft rock reconstituted soils). Each type of reconstructed soil formed a treatment and there were 3 repetitions for each treatment, totalling 12 small test plots for the long-term positioning test. The dosage of meteorite, shale, sand, and soft rock was 10^–3^ m^3^/m^2^. The dimensions of all small test plots were 10 × 20 m^2^. To observe soil respiration, one soil respiration ring with an inner diameter of 10 cm was buried in each small test plot, extending 2 cm above ground. The vegetation in the test plots consisted of tall fescue (*Festuca arundinacea*). All vegetation (excluding roots) was removed from the soil within the respiration rings to ensure no vegetative growth in the rings during the entire observation period. The physical and chemical properties of the test plots are shown in Table 1. One 2 m × 2 m rectangular square was randomly set as a root exclusion test plot in each small test plot. One soil respiration ring of the same specification was embedded in each root exclusion treatment plot to observe heterotrophic respiration in the reconstructed soils. A 40-cm deep small ditch was excavated around the root exclusion treatment plots, lined with asbestos sheets, and back-filled with soil to the level of the section. The value of autotrophic respiration was obtained by subtracting heterotrophic respiration from soil respiration.

**Table 1:** Basic physical and chemical properties of four reconstructed soils at 0 ~ 20 cm depth.

Detection indicator	Reconstituted soil mass types			
	Meteorite	Shale	Sand	Soft rock
pH	8.55	8.49	8.51	8.49
Organic carbon (g kg^−1^)	3.41	3.75	3.7	4.77
Total nitrogen (g kg^−1^)	0.56	0.36	0.44	0.48
Available phosphorus (mg kg^−1^)	12.93	26.33	27.27	21.7
Available potassium (mg kg^−1^)	136.96	130.15	115.54	111.65
**Size grading**				
< 0.002 mm	16.47	16.88	15.17	17.85
0.002–0.05 mm	79.87	76.09	79.99	79.22
> 0.05 mm	6.04	7.03	4.84	2.93

### Research methods

Soil respiration of all the test plots was measured on three typical days from 9:30 to 11:00 (clear weather, no precipitation three days ago, wind speed less than 0.5 m/s) each month from November 2017 to October 2018. Soil respiration measurements were performed using a soil carbon flux measurement system (LI-8100, LI-COR Biosciences, Lincoln, NE, USA) equipped with a survey chamber of 20 cm in diameter and equipped with an auxiliary sensor connected to the main unit^17–19^. The sensor can be connected to up to 4 thermocouples (3 input voltages and 1 soil water content channel). When measuring soil respiration, the respiration chamber was placed on the buried soil respiration ring to ensure that the connection between the respiration ring and the soil respiration chamber was sealed, and the electronic temperature probe connected to the sensor. Moreover, the time domain reflectometry probes were inserted vertically into the soil near each respiration ring to measure soil carbon flux, soil temperature at 5 cm, and water content at 10 cm. Each soil respiration ring was measured 3 times and the measurement time was 4 min each time.

### Data analyses

One-way ANOVA was used to analyse differences in soil temperature, soil volumetric water, and soil respiration of four reconstituted soils. All statistical tests were conducted with the SPSS software (version 22.0; SPSS Inc., Chicago, IL, USA). Nonlinear regression was used to assess the relationship between soil respiration and hydrothermal influence factors of four reconstructed soils, and Q_10_ was estimated. The relationships between soil respiration and soil temperature and water content were fitted by an exponential model (1) and a quadratic curve model (2), respectively. Additionally, the relationship between soil respiration and both soil temperature and soil volumetric water content was fitted by power-index model (3)^20^:

1 $$R_{S} = \, a\;{\text{e}}^{bT}$$

2 $$R_{{\text{S}}} = a\;{\text{w}}^{2} + b\;{\text{w }} + c$$

3 $$R_{{\text{S}}} = a\;{\text{e}}^{{b_{{\text{T}}} }} \;{\text{w}}^{c}$$

where R_S_ is the soil respiration rate (μmol m^−2^ s^−1^); T is the soil temperature (°C); w is the soil volumetric water content (%); *a*, *b*, and *c* are the model parameters.

## Results

### Seasonal changes in respiration and hydrothermal factors of reconstructed soils

#### Seasonal variation in soil temperature and volumetric water content

**Figure 1:**
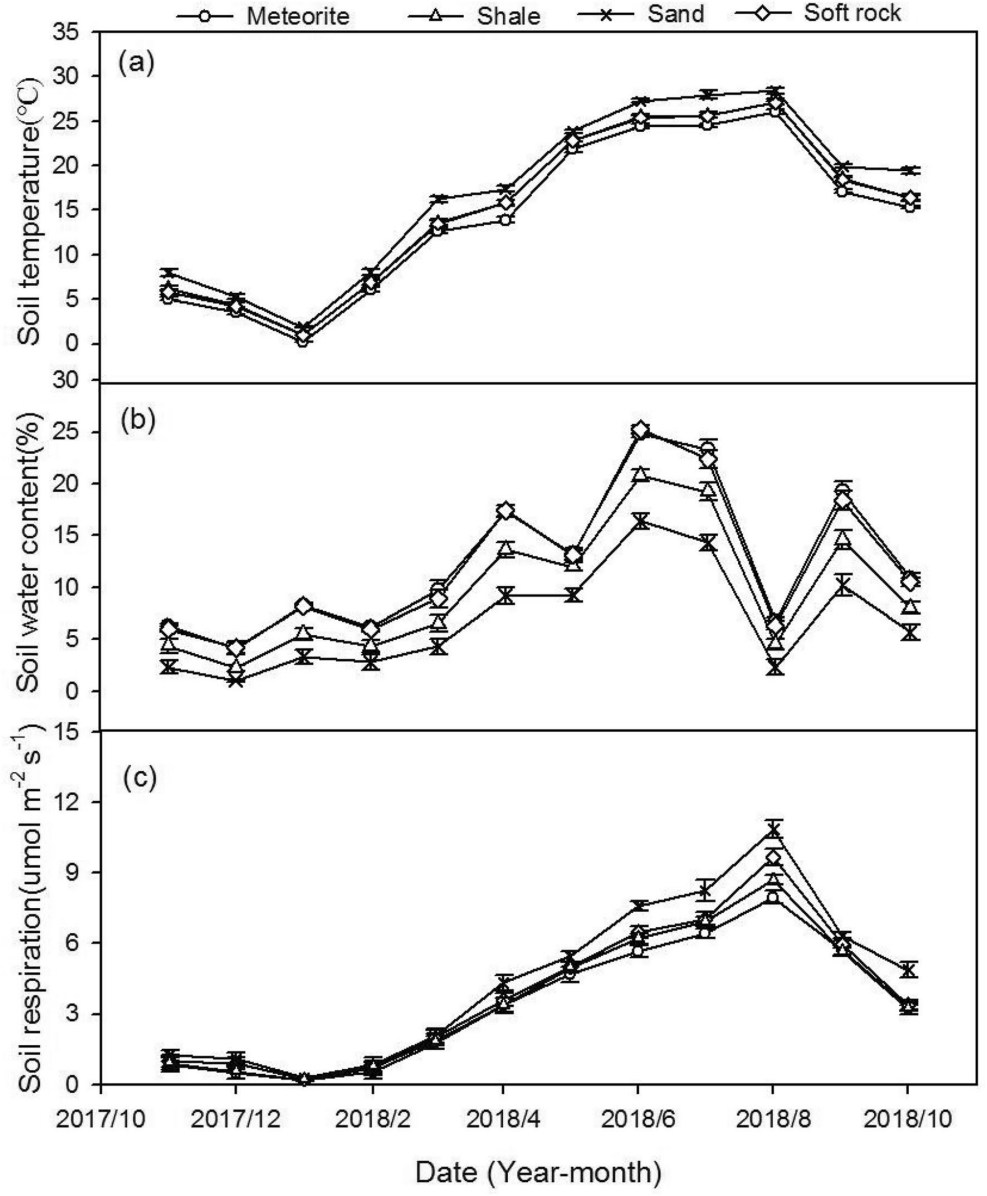
The seasonal change trend of temperature (**a**), volumetric water content (**b**) and respiration rate (**c**) of the four reconstructed soils.

The monthly mean soil temperature of the four reconstructed soils was highest in summer and lowest in winter; the maximum and minimum values were measured in August 2018 and January 2018, respectively (Fig. 1a). The monthly mean temperature of the sand or meteorite reconstructed soils was significantly different from that of the other three (*p* < 0.05). The monthly average soil temperature of the reconstructed soil with addition of shale and meteorite was not significantly different (*p* > 0.05). The order of the monthly average soil temperature of the four reconstructed soils throughout the year was sand > shale > soft rock > meteorite (Fig. 1a). The maximum and minimum monthly average soil volumetric water content of the four reconstructed soils throughout the year appeared in June 2018 and December 2017, respectively. The monthly average volumetric water content of the reconstructed soils with addition of soft rock and vermiculite was not significant (*p* > 0.05). The monthly average soil volumetric water content of the shale or sand reconstructed soils was significantly different from that of the other three (*p* < 0.05) (Fig. 1b).

#### Seasonal variation in soil respiration

The monthly average soil respiration rate increased gradually with increasing soil temperature, exhibiting obvious seasonal changes—the highest was detected in summer and the lowest in winter. Throughout the year, the monthly average soil respiration rate of the four reconstructed soils was in the range of 0.16–7.97 μmol m^−2^ s^−1^ (meteorites), 0.21–9.69 μmol m^−2^ s^−1^ (shale), 0.26–10.87 μmol m^−2^ s^−1^ (sand), and 0.20–8.71 μmol m^−2^ s^−1^ (soft rock). The monthly mean soil respiration rate of the four reconstructed soils was highest in summer and lowest in winter; the maximum and minimum values were measured in August 2018 and January 2018, respectively. The maximum and minimum values of soil respiration and temperature change of the four reconstructed soils were the same month. In winter, the monthly average respiration rates of the four reconstructed soils were not significantly different (*p* > 0.05). In summer, the monthly average respiration rate of the sand or meteorite reconstructed soils was significantly different from that of the other three (*p* < 0.05). In spring, a significantly different monthly average respiration rate was detected between all reconstructed soils in March (*p* > 0.05), but only between the sand reconstructed soils and other reconstructed soils in April and May (*p* < 0.05). In autumn, the difference remained significant between sand reconstructed soils and other reconstructed soils in September and October (*p* < 0.05). In November, the monthly average respiration rate of the four reconstructed soils showed a significant difference (*p* > 0.05) (Fig. 1c).

### The relationship between respiration and hydrothermal factors of reconstructed soils

#### The effect of seasonal variation on the relationship between soil respiration and soil temperature

The relationship between soil respiration and soil temperature at 5 cm depth of the four reconstructed soils can be characterised by an exponential model (Table 2). There was a significant positive exponential correlation between respiration rate and temperature of the four reconstructed soils. The soil temperature of meteorite, shale, sand, and soft rock reconstructed soils accounted for 82%, 92%, 86%, and 98% of seasonal variation, respectively, in soil respiration in winter; 77%, 99%, 88%, and 97% of seasonal variation, respectively, in soil respiration in spring; 83%, 97%, 99%, and 92% of seasonal variation, respectively, in soil respiration in summer; and 88%, 86%, 93%, and 84% of seasonal variation, respectively, in soil respiration in autumn.

**Table 2:** Relationship between soil respiration rate and temperature for reconstituted soils.

Season	Reconstituted soil mass types	Index model	R^2^	*p*
Winter	Meteorite	*R*_S_ = 0.1951e^0.4374*T*S^	0.82	0.001
	Shale	*R*_S_ = 0.1708e^0.3115*T*S^	0.92	0.001
	Sand	*R*_S_ = 0.1874e^0.2618*T*S^	0.86	0.001
	Soft rock	*R*_S_ = 0.1481e^0.3104*T*S^	0.98	0.001
Spring	Meteorite	*R*_S_ = 6.0306e^0.2800*T*S^	0.77	0.001
	Shale	*R*_S_ = 0.1829e^0.1828*T*S^	0.99	0.001
	Sand	*R*_S_ = 0.2350e^0.1522*T*S^	0.88	0.001
	Soft rock	*R*_S_ = 0.2400e^0.1604*T*S^	0.97	0.001
Summer	Meteorite	*R*_S_ = 16.038e^0.0689*T*S^	0.83	0.001
	Shale	*R*_S_ = 0.4036e^0.1103*T*S^	0.97	0.001
	Sand	*R*_S_ = 0.4908e^0.1008*T*S^	0.99	0.001
	Soft rock	*R*_S_ = 0.4450e^0.1056*T*S^	0.92	0.001
Autumn	Meteorite	*R*_S_ = 10.177e^0.1104*T*S^	0.88	0.001
	Shale	*R*_S_ = 0.9686e^0.0864*T*S^	0.86	0.001
	Sand	*R*_S_ = 1.1798e^0.0784*T*S^	0.93	0.001
	Soft rock	*R*_S_ = 1.0333e^0.0803*T*S^	0.84	0.001

#### The effect of seasonal variation on the relationship between soil respiration and volumetric water content

The relationship between soil respiration and volumetric water content of the four reconstructed soils can be characterised by a quadratic curve model. There was a significant negative correlation between the two, but the ability to interpret was significantly lower than that of temperature (Table 3). Although the changes in soil temperature accounted for most seasonal changes in soil respiration, the temperature effects were not always consistent. Soil respiration was affected by other factors, such as volumetric water content. Thus, volumetric water content at 0–10 cm depth was determined to be the key environmental factor of the soil respiration changes.

**Table 3:** Relationship between soil respiration and volumetric water content for reconstituted soils.

Season	Reconstituted soil mass types	Quadratic model	R^2^	*p*
Winter	Meteorite	*R*_S_ = − 0.0531 + 0.5778*W*_S_ − 0.9956*W*_S_^2^	0.42	0.001
	Shale	*R*_S_ = − 0.0902 + 0.4885*W*_S_ − 0.2519*W*_S_^2^	0.36	0.001
	Sand	*R*_S_ = − 1.8117 + 9.4380*W*_S_ − 12.496*W*_S_^2^	0.26	0.001
	Soft rock	*R*_S_ = − 0.4681 + 6.4734*W*_S_ − 12.182*W*_S_^2^	0.21	0.001
Spring	Meteorite	*R*_S_ = − 0.1501 + 4.2643*W*_S_ − 25.456*W*_S_^2^	0.39	0.001
	Shale	*R*_S_ = − 0.2014 + 4.2757*W*_S_ − 17.296*W*_S_^2^	0.54	0.001
	Sand	*R*_S_ = − 15.297 + 20.626*W*_S_ − 14.9123*W*_S_^2^	0.36	0.001
	Soft rock	*R*_S_ = − 0.1158 + 3.1704*W*_S_ − 18.286*W*_S_^2^	0.43	0.001
Summer	Meteorite	*R*_S_ = − 0.0221 + 0.5719*W*_S_ − 5.1263*W*_S_^2^	0.23	0.001
	Shale	*R*_S_ = − 0.0115 + 0.0939*W*_S_ − 9.5003*W*_S_^2^	0.37	0.001
	Sand	*R*_S_ = − 0.0694 + 1.0969*W*_S_ − 6.1039*W*_S_^2^	0.46	0.001
	Soft rock	*R*_S_ = − 0.0202 + 0.5355*W*_S_ − 4.3636*W*_S_^2^	0.52	0.001
Autumn	Meteorite	*R*_S_ = − 0.0174 + 0.8185*W*_S_ − 3.5937*W*_S_^2^	0.48	0.001
	Shale	*R*_S_ = − 0.0254 + 0.9676*W*_S_ − 7.7416*W*_S_^2^	0.29	0.001
	Sand	*R*_S_ = − 0.0623 + 2.0134*W*_S_ − 3.0939*W*_S_^2^	0.52	0.001
	Soft rock	*R*_*S*_ = − 0.0538 + 1.9411*W*_S_ − 8.7895*W*_S_^2^	0.51	0.001

#### Seasonal variation in soil respiration, soil temperature, and volumetric water content

The effect of seasonal variation on the correlation of soil respiration with soil temperature and volumetric water content can be characterised by a power-exponential model (Table 4). In winter, soil temperature and volumetric water content of the four reconstructed soils explained 88% (meteorite), 93% (shale), 92% (sand), and 98% (soft rock) of seasonal changes in soil respiration; in spring, they explained 86% (meteorite), 98% (shale), 94% (sand), and 98% (soft rock) of seasonal changes in soil respiration; in summer, soil temperature and volumetric water content explained 90% (meteorite), 98% (shale), 98% (sand), and 95% (soft rock) of seasonal changes in soil respiration; and in autumn, they explained 90% (meteorite), 91% (shale), 97% (sand), and 92% (soft rock) of seasonal changes in soil respiration.

**Table 4:** Relationship between soil respiration rate and hydrothermal factors for reconstituted soils.

Season	Reconstituted soil mass types	Power-exponential model	R^2^	*p*
Winter	Meteorite	*R*_S_ = 3.843e^1.838*T*S^*W*_S_^0.171^	0.88	0.001
	Shale	*R*_S_ = 4..655e^1.600*T*S^*W*_S_^0.919^	0.93	0.001
	Sand	*R*_S_ = 3.335e^0.954*T*S^*W*_S_^0.151^	0.92	0.001
	Soft rock	*R*_S_ = 3.257e^0.879*T*S^*W*_S_^0.402^	0.98	0.001
Spring	Meteorite	*R*_S_ = 0.082e^0.074*T*S^*W*_S_^0.942^	0.86	0.001
	Shale	*R*_S_ = 0.278e^0.059*T*S^*W*_S_^0.621^	0.98	0.001
	Sand	*R*_S_ = 0.304e^0.035*T*S^*W*_S_^0.924^	0.94	0.001
	Soft rock	*R*_S_ = 0.154e^0.081*T*S^*W*_S_^0.632^	0.98	0.001
Summer	Meteorite	*R*_S_ = 5.671e^0.027*T*S^*W*_S_^0.185^	0.90	0.001
	Shale	*R*_S_ = 1.426e^0.079*T*S^*W*_S_^0.159^	0.98	0.001
	Sand	*R*_S_ = 1.121e^0.084*T*S^*W*_S_^0.133^	0.98	0.001
	Soft rock	*R*_S_ = 2.174e^0.061*T*S^*W*_S_^0.143^	0.95	0.001
Autumn	Meteorite	*R*_S_ = 0.142e^0.094*T*S^*W*_S_^0.704^	0.90	0.001
	Shale	*R*_S_ = 0.224e^0.080*T*S^*W*_S_^0.671^	0.91	0.001
	Sand	*R*_S_ = 0.433e^0.091*T*S^*W*_S_^0.374^	0.97	0.001
	Soft rock	R_S_ = 0.163e^0.085*T*S^*W*_S_^0.677^	0.92	0.001

### Seasonal changes in respiration and its components of reconstructed soils

#### Seasonal variation in respiration components

The rates of heterotrophic respiration and autotrophic respiration of the four reconstituted soils were highest in summer and lowest in winter. The maximum and minimum values appeared in August and January, respectively. The rates of heterotrophic and autotrophic respiration of the four reconstructed soils showed a statistically significant difference (*p* < 0.05). In January, the heterotrophic respiration of the four reconstructed soils had the highest monthly mean contribution rate to total respiration, and autotrophic respiration had the minimal. In June, the reconstructed soils with added meteorites, shale, and soft rock had the highest monthly mean contribution rate to total respiration, and the lowest contribution rate of autotrophic respiration. In July, the highest monthly contribution rate to total respiration and the lowest autotrophic respiration was measured in sand reconstructed soils.

#### The contribution of respiration components to total soil respiration of four reconstructed soils

**Figure 2:**
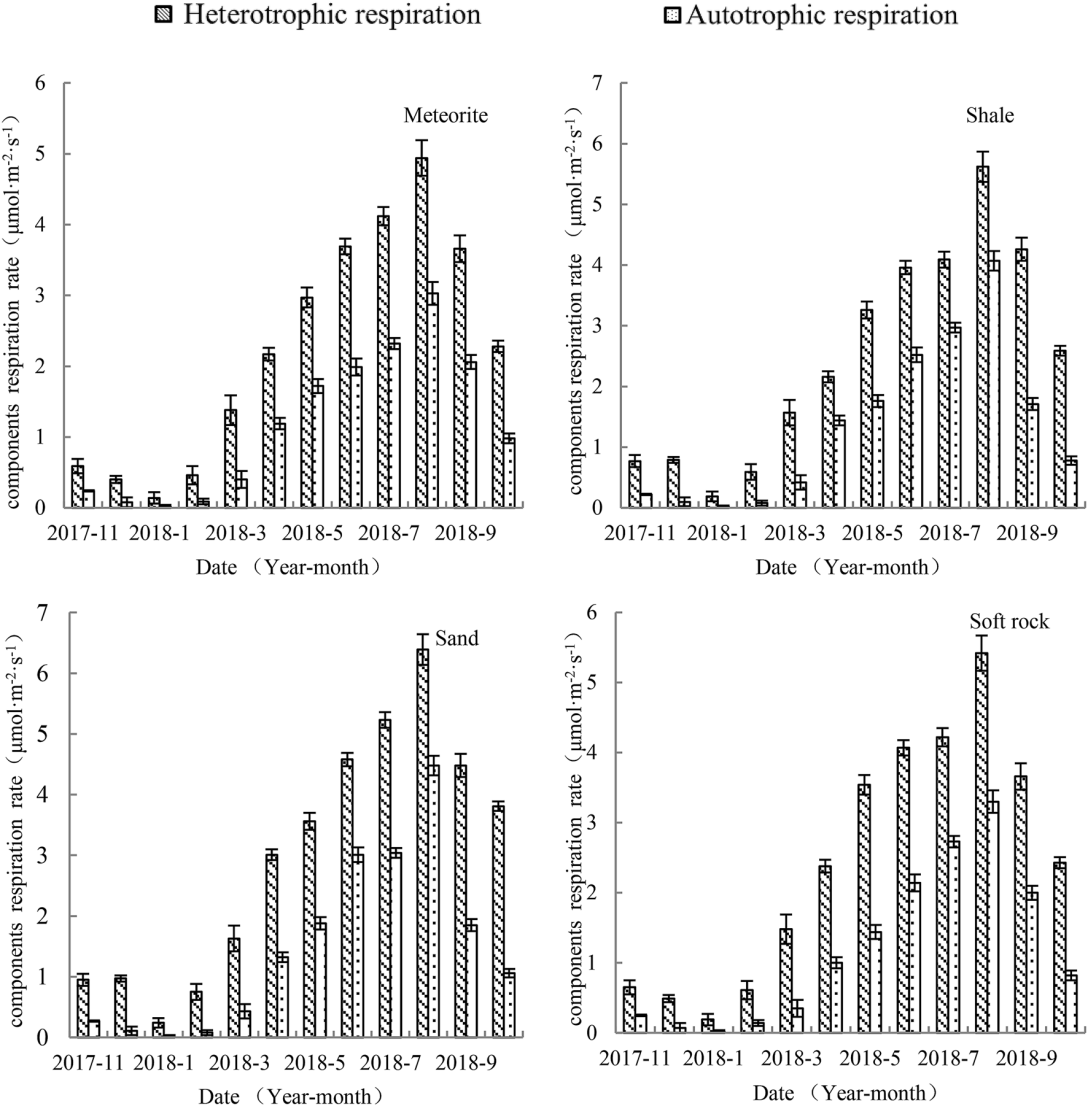
Components respiration rate of for different reconstructed soils.

The largest contribution rate of heterotrophic respiration and the smallest contribution rate of autotrophic respiration to total respiration for the four reconstructed soils were measured in January. The largest monthly average contribution rate of heterotrophic respiration rate to total soil respiration and the smallest autotrophic respiration rate were identified for meteorite, shale, and soft rock reconstructed soils in June and for sand reconstructed soils in July (Fig. 2).

In winter, the monthly average contribution rate of heterotrophic respiration to total respiration of the reconstructed soils with meteorite, shale, sand, and soft rock was 83.3–87.5%, 88.1–90.5%, 89.8–92.3%, and 81.3–95.0%, respectively, and the respective monthly average contribution rate of autotrophic respiration to total respiration was 12.5–16.7%, 9.5–11.9%, 7.7–10.2% and 5.0–18.7%, respectively. In spring, the monthly average contribution rate of heterotrophic respiration to total respiration was in the range of 63.3–73.3% (meteorite), 60.0–79.0% (shale), 65.4–79.1% (sand), and 65.5–71.7% (soft rock reconstructed soils). The monthly average contribution rate of autotrophic respiration of the four reconstructed soils was 22.7–36.7% (meteorite), 21.0–35.1% (shale), 20.9–34.6% (sand), 19.1–29.6% (soft rock). In summer, the monthly average contribution rate of heterotrophic respiration to total respiration of the four reconstructed soils was 62.0–65.0% (meteorite), 58.0–61.1% (shale), 58.8–63.3% (sand), and 60.7–65.5% (soft rock). The monthly average contribution rate of autotrophic respiration to total respiration of the four reconstructed soils was 35.0–38.0% (meteorite), 38.9–42.0% (shale), 36.7–41.2% (sand), and 35.5–39.3% (soft rock). In autumn, the monthly average contribution rate of heterotrophic respiration to total respiration of the four reconstructed soils was 71.7–64.0% (meteorite), 77.8–71.4% (shale), 70.8–78.2% (sand), and 64.7–74.8% (soft rock). The monthly average contribution rate of autotrophic respiration to total respiration of the four reconstructed soils was 28.3–36.0% (meteorite), 22.2–28.6% (shale), 21.8–29.2% (sand), and 25.2–35.3% (soft rock) (Table 5).

**Table 5:** Monthly average contribution of soil respiration components to total respiration for different reconstituted soils.

Season	Month	Soil respiration type	Monthly average contribution rate (%)			
			Meteorite	Shale	Sand	Soft rock
Winter	2017-12	R_H_	83.3	88.8	89.8	87.5
		R_A_	16.7	11.2	10.2	12.5
	2018-1	R_H_	87.5	90.5	92.3	95.0
		R_A_	12.5	9.5	7.7	5.0
	2018-2	R_H_	83.6	88.1	90.4	81.3
		R_A_	16.4	11.9	9.6	18.7
Spring	2018-3	R_H_	77.3	79.0	79.1	80.9
		R_A_	22.7	21.0	20.9	19.1
	2018-4	R_H_	64.6	60.0	69.5	70.4
		R_A_	35.4	40.0	30.5	29.6
	2018-5	R_H_	63.3	64.9	65.4	71.1
		R_A_	36.7	35.1	34.6	28.9
Summer	2018-6	R_H_	65.0	61.1	60.3	65.5
		R_A_	35.0	38.9	39.7	35.5
	2018-7	R_H_	64.0	58.0	63.3	60.7
		R_A_	36.0	42.0	36.7	39.3
	2018-8	R_H_	62.0	58.0	58.8	62.2
		R_A_	38.0	42.0	41.2	37.8
Autumn	2018-9	R_H_	64.0	71.4	70.8	64.7
		R_A_	36.0	28.6	29.2	35.3
	2018-10	R_H_	69.9	76.9	78.2	74.8
		R_A_	30.1	23.1	21.8	25.2
	2017-11	R_H_	71.7	77.8	77.9	72.0
		R_A_	28.3	22.2	22.1	28.0

*R*_*H*_ heterotrophic respiration, *R*_*A*_ autotrophic respiration.

## Discussion

Different soil constituents have different properties, and their effects on seasonal changes in soil respiration and hydrothermal factors vary. The results of this study show that the soil temperature of the sand reconstituted soil was the highest each month, and the soil temperature of the reconstituted soil was the lowest in each month. It mainly is due to the difference in specific heat capacity. Meteorite has good water storage and moisture retention properties. Sand heats up faster, thereby approaching the temperature of the environment faster. Once the temperature of the sand is consistent with the ambient temperature, heat conduction will no longer occur. In contrast, meteorite has a large specific heat capacity; therefore, it takes a long time to raise the temperature, while sand cools quickly. Soft rock and shale have a high content of clay particles, which are soil-forming materials. Meteorite can increase soil water retention and water occupies pores in the soil, resulting in relatively low CO_2_ content in the soil and hence soil respiration, which is good for environmental protection. At the same time, sand, another soil improvement material, can increase the air content in soil and thus increase the intensity of soil respiration, which is not conducive to environmental protection. During land remediation, local governments need to make targeted choices based on the characteristics of various soil materials. However, the respiration of the sand reconstituted soil was only different from other reconstituted soils in the summer, to ensure a good regional ecological environment; they need to formulate special carbon emission reduction measures, especially during the summer.

Numerous studies have suggested that soil temperature can explain seasonal changes in soil respiration^21,22^. In the present study, the relationship between seasonal changes in respiration and temperature of the four reconstructed soils was significantly exponentially correlated, which was consistent with other studies. During the monitoring period, the soil volumetric water content was lower than the field water holding capacity and there was no inhibition of soil respiration. Thus, soil temperature explained the seasonal changes in soil respiration. The relationship between soil respiration and soil moisture is generally linear or characterised by quadratic curves^23–25^. The correlation between soil respiration and soil volumetric water content was weaker than between soil respiration and soil temperature. Soil respiration and soil volumetric water content was characterised by a quadratic curve. Soil moisture is considered a major factor of seasonal changes in soil respiration when compared with temperature in tropical forests^21^. The effect of soil moisture on soil respiration was complex. The changes in the relationship between soil respiration and volumetric water content may have been caused by the variation in precipitation, annual precipitation, and soil structure in the study area. There was a threshold value at which soil moisture affected soil respiration; this threshold value was affected by soil texture and structure. Different variables were used to describe the threshold of soil moisture; for example, this threshold was defined as the lower limit of water threshold^26,27^, as 20% of soil moisture and the soil water potential of 1500 kPa^28^, and by the field water holding capacity^29^. Wilting point and 1/3 of the field water holding capacity have been used as the upper soil moisture threshold value^30^. Therefore, the changes in threshold value of the volumetric water content and their effect on soil respiration was the focus of the next step.

Water and temperature as environmental factors, along with crop biological characteristics and human factors, are the main factors affecting soil respiration in farmland ecosystems in China^31^. The most important environmental factors for seasonal changes in soil respiration are soil temperature and moisture. The two-factors model of soil respiration, as one factor, and temperature and moisture, as the other factor, can further improve the accuracy of predicting soil respiration^32,33^. However, in the present study, the results were slightly different from the predicted values due to differences in environmental factors such as study area, sampling time, and ecosystem type. The mainstream view is that soil temperature and soil moisture can better explain the seasonal changes in soil respiration^34,35^. In the present study, the effect of soil volumetric water content on soil respiration was weak, but it could not be neglected. Therefore, a two-factors model, implementing soil temperature and moisture as one factor and soil respiration as the other factor, was fitted, with coefficient of determination mostly greater than that of the single factor model based on temperature. This indicated that the seasonal changes in soil temperature and soil volume and water content of the four reconstituted soils are the most important environmental factors causing seasonal changes in soil respiration. The two-factors variable model can thus be used for predicting the seasonal variations in soil respiration in reconstructed soils of the study area. Soil respiration can be explained by the two-factors relationship equations, but other studies that the factors affecting soil respiration are inherently complex and difficult to distinguish in field conditions. Therefore, the independent role of single factors in determining soil temperature and soil moisture warrants further study.

The effects of environmental factors on soil respiration include not only direct effects, but also indirect effects. Indirect impact refers to the impact of environmental factors on the biological and ecological processes of soil and crops, and the indirect impact on soil respiration. For example, temperature changes lead to changes in soil microbial communities and activities which indirectly affect soil respiration^36^. The indicators of soil microbial changes mainly include soil bacterial biomass, total surface area and specific surface area of soil bacteria, total number of soil bacteria, average soil cell volume, enzyme activity and metabolic entropy, etc.^37^; temperature and moisture changes lead to the occurrence of soil physical and chemical properties and biological factors Change^38^, the indicators of the change of soil physical properties mainly include soil bulk density, soil mechanical composition, field water holding capacity, etc^39^. The indicators of the change of soil chemical properties mainly include soil organic matter, soil water-soluble organic carbon, soil easily oxidisable organic carbon and soil pH, etc^40,41^. The indicators of changes in biological factors mainly include vegetation coverage index, leaf area index, etc^42,43^. By measuring these indicators, the relationship between each indicator and the soil respiration rate of the reconstructed soils can be analysed, the main factors affecting soil respiration can be judged, the driving mechanism of the reconstructed soil respiration can be clarified, and reasonable carbon emission reduction measures can be formulated; Dynamic monitoring of soil respiration quickly characterises the changes in the quality of the reconstructed soil, and formulates reasonable soil improvement and crop planting plans. Therefore, it is urgent to carry out research on the relationship between soil microorganisms, physical and chemical properties, and biological factors and respiration.

## Conclusion

1. The monthly average respiration rate and the rates of its components in the four reconstructed soils were the highest in summer and lowest in winter. In winter, the monthly average respiration rates of the four reconstructed soils were not different (*p* > 0.05). In summer, the monthly average respiration rate of the sand or meteorite reconstructed soil was different from that of the other three (*p* < 0.05).
2. The heterotrophic and autotrophic respiration rates were different between the four reconstructed soils (*p* < 0.05). The contribution of heterotrophic respiration to total respiration in the four reconstructed soils was greater than that of autotrophic respiration throughout the year. In winter, autotrophic respiration accounts for the smallest proportion of total respiration. As the temperature rises, the proportion of autotrophic respiration to total respiration gradually increases and peaks in summer. In summer, the proportion of heterotrophic respiration of the total respiration is the smallest. With the decrease in temperature, the proportion of heterotrophic respiration of the total respiration gradually increases and peaks in winter.
3. The maximum and minimum values of the monthly average respiration rate of the four reconstructed soils coincided with the months with maximum and minimum soil temperature. The soil volumetric water content changed with the amount of precipitation. The correlation between soil respiration and temperature was greater than that between soil respiration and volumetric water content.
4. The correlation in seasonal variation between respiration of the four remodelled soils and hydrothermal factors can be characterised by an exponential function and power-exponential function in the study area.
